# Dual targeting of MDM2 and BCL2 as a therapeutic strategy in neuroblastoma

**DOI:** 10.18632/oncotarget.18982

**Published:** 2017-07-04

**Authors:** Alan Van Goethem, Nurten Yigit, Myrthala Moreno-Smith, Sanjeev A. Vasudevan, Eveline Barbieri, Frank Speleman, Jason Shohet, Jo Vandesompele, Tom Van Maerken

**Affiliations:** ^1^ Center for Medical Genetics Ghent (CMGG), Ghent University, Ghent, Belgium; ^2^ Cancer Research Institute Ghent (CRIG), Ghent University, Ghent, Belgium; ^3^ Department of Pediatrics, Section of Hematology-Oncology, Texas Children’s Cancer Center, Baylor College of Medicine, Houston, Texas, USA; ^4^ Bioinformatics Institute Ghent (BIG), Ghent University, Ghent, Belgium

**Keywords:** neuroblastoma, idasanutlin, p53, venetoclax, synergism

## Abstract

Wild-type p53 tumor suppressor activity in neuroblastoma tumors is hampered by increased MDM2 activity, making selective MDM2 antagonists an attractive therapeutic strategy for this childhood malignancy. Since monotherapy in cancer is generally not providing long-lasting clinical responses, we here aimed to identify small molecule drugs that synergize with idasanutlin (RG7388). To this purpose we evaluated 15 targeted drugs in combination with idasanutlin in three p53 wild type neuroblastoma cell lines and identified the BCL2 inhibitor venetoclax (ABT-199) as a promising interaction partner. The venetoclax/idasanutlin combination was consistently found to be highly synergistic in a diverse panel of neuroblastoma cell lines, including cells with high MCL1 expression levels. A more pronounced induction of apoptosis was found to underlie the synergistic interaction, as evidenced by caspase-3/7 and cleaved PARP measurements. Mice carrying orthotopic xenografts of neuroblastoma cells treated with both idasanutlin and venetoclax had drastically lower tumor weights than mice treated with either treatment alone. In conclusion, these data strongly support the further evaluation of dual BCL2/MDM2 targeting as a therapeutic strategy in neuroblastoma.

## INTRODUCTION

Neuroblastoma, the second most common solid tumor in childhood, is characterized by a heterogeneous clinical behavior, ranging from spontaneous regression to aggressive and inexorable disease [1]. Fifty percent of cases are considered high risk, with long-term survival rates after multimodal chemo- and immunotherapy below 40% [2]. Like many childhood cancers, mutations in the *TP53* tumor suppressor gene are rarely encountered in primary neuroblastoma tumors (< 2%), and remain rather uncommon at relapse (< 15%) [3, 4]. However, neuroblastoma cells with wild-type *TP53* almost invariably harbor defects in other components of the TP53 pathway. In particular, amplification and increased expression of *MDM2* and suppression of CDKN2A (p14^ARF^) have been reported. These abnormalities converge into the central CDKN2A/MDM2/TP53 axis resulting in increased activity of MDM2, the principal TP53 inhibitor, and subsequent impairment of normal TP53 functioning [5]. The importance of MDM2-mediated suppression of the TP53 pathway in neuroblastoma has convincingly been demonstrated *in vivo* using an *MDM2*-haploinsufficient mouse model in which tumor latency was delayed while tumor incidence and growth were remarkably reduced [6].

These biological conditions appear to make neuroblastoma cells highly sensitive to treatment with MDM2 antagonists. Indeed, the anti-tumor effects of nutlin-3, a *cis*-imidazoline analogue that selectively disrupts the interaction between TP53 and MDM2 resulting in targeted activation of the TP53 pathway, have extensively been reported in neuroblastoma [7–11]. Treatment with nutlin-3 resulted in nuclear accumulation of TP53, transcriptional activation of TP53 target genes and subsequent induction of cell cycle arrest and apoptosis both *in vitro* and *in vivo*. While initial studies in neuroblastoma have been performed using the original lead compound, nutlin-3, second-generation inhibitors with superior potency and selectivity, such as RG7388 (idasanutlin), are now prioritized for clinical development [12, 13]. Investigation of the clinical trial drug idasanutlin in preclinical model systems of neuroblastoma is still limited. Successful introduction of targeted therapeutics in the clinic requires the identification of well-matched drugs for combination therapy to increase therapeutic efficacy and to prevent the development of treatment resistance. Resistance of neuroblastoma cells to nutlin-3 was found to be mainly mediated by the acquisition of *de novo* mutations in the TP53 DNA binding domain and to result in a multi-drug resistant phenotype [14]. In several cancer entities, experimental drug combinations with nutlin-3 have been investigated [15, 16]. In neuroblastoma, reports have mainly been limited to chemotherapeutics that enhance the efficacy of MDM2 antagonists [8, 17–19]. To identify targeted drugs that could potentiate the anti-tumor effects of idasanutlin, we evaluated 15 targeted drugs in combination with idasanutlin in neuroblastoma cells. The highest degree of synergism was consistently observed if idasanutlin was combined with the BCL2/BCL-XL inhibitor ABT-263. A comparable strong synergistic activity was recorded if idasanutlin was combined with the more selective BCL2 inhibitor ABT-199 (venetoclax), irrespective of basal BCL2 and MCL1 expression levels. In neuroblastoma cells, the combination therapy resulted in increased induction of apoptosis compared to either treatment alone, consistent with the proposed mechanism of action. In addition, when administered to mice carrying orthotopic xenografts of human neuroblastoma cells, the combination of idasanutlin and venetoclax was significantly more effective than either treatment alone.

## RESULTS

### Combining ABT-263 and idasanutlin is highly synergistic in neuroblastoma cells

**Figure 1:**
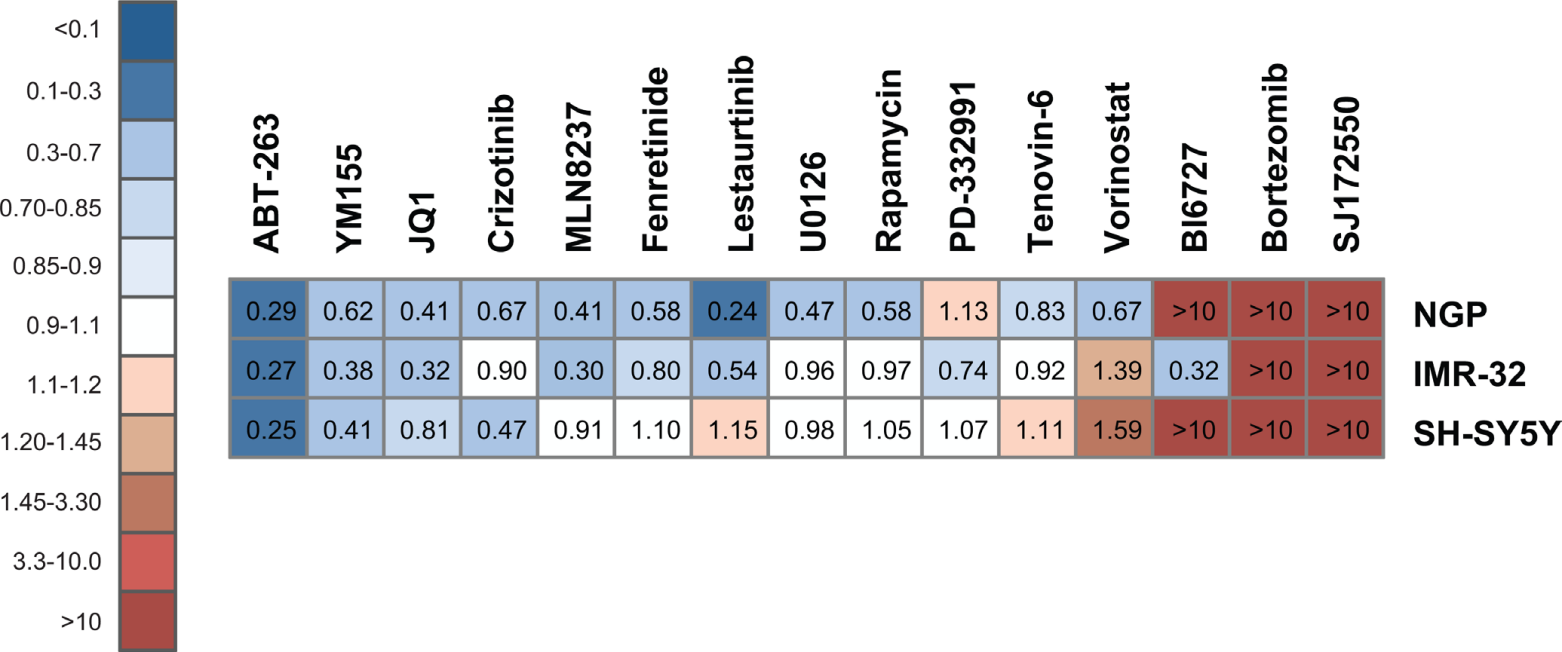
Heatmap with CI values of 15 targeted drugs combined with idasanutlin in NGP, IMR-32 and SH-SY5Y cells Cell viability and CI values were determined 24h after treatment with idasanutlin and one of the selected compounds. Depicted CI values represent the average of the CIs at ED50, ED75 and ED90 of at least two independent experiments. > 10: very strong antagonism; 3.30–10: strong antagonism; 1.45–3.30: antagonism; 1.20–1.45: moderate antagonism; 1.10–1.20: slight antagonism; 0.90–1.10: nearly additive; 0.85–0.90: slight synergism; 0.70–0.85: moderate synergism; 0.30–0.70: synergism; 0.10–0.30: strong synergism; < 0.10: very strong synergism. Not for all compounds these effective doses could be achieved within a therapeutic range. For SK-N-BE(2c) no CI values could be calculated.

We selected three neuroblastoma cell lines with wild-type *TP53* (SH-SY5Y, IMR-32 and NGP) and used one *TP53* mutant cell line (SK-N-BE(2c)) as a negative control. We evaluated combination treatment of idasanutlin with 15 other targeted drugs, selected on their ability to target pathways that are considered important in neuroblastoma biology (Supplementary Table 1 provides a detailed overview of used compounds and concentration ranges). We exposed neuroblastoma cells to a concentration series of either idasanutlin or one of the other compounds or a combination of both for 24 hours after which cell viability and CI values were determined (Figure 1). In all three cell lines with wild-type *TP53*, we found that the combination of the BCL2/BCL-XL antagonist ABT-263 (navitoclax) with idasanutlin consistently resulted in highly synergistic CI values: 0.29, 0.27 and 0.25 for NGP, IMR-32 and SH-SY5Y, respectively. In contrast, we did not observe any effects in SK-N-BE(2c) cells using the same dose range (Supplementary Table 2). These results point to a highly synergistic interaction between idasanutlin and ABT-263 in neuroblastoma cells. Furthermore, we found YM155, a small molecule that inhibits the transactivation of the anti-apoptotic protein survivin (*BIRC5*), to potently enhance the efficacy of idasanutlin in the *TP53* wild-type neuroblastoma cells, with CI values of 0.62, 0.38 and 0.41 for NGP, IMR-32 and SH-SY5Y, respectively. The anti-apoptotic BCL-XL, BCL2 and survivin proteins have been described to counteract TP53-mediated apoptosis and inhibition of these proteins has been shown to lower the threshold for apoptosis and to enhance the efficacy of TP53 reactivation therapies [20, 21].

### Venetoclax and idasanutlin are highly synergistic in human neuroblastoma cell lines

**Figure 2:**
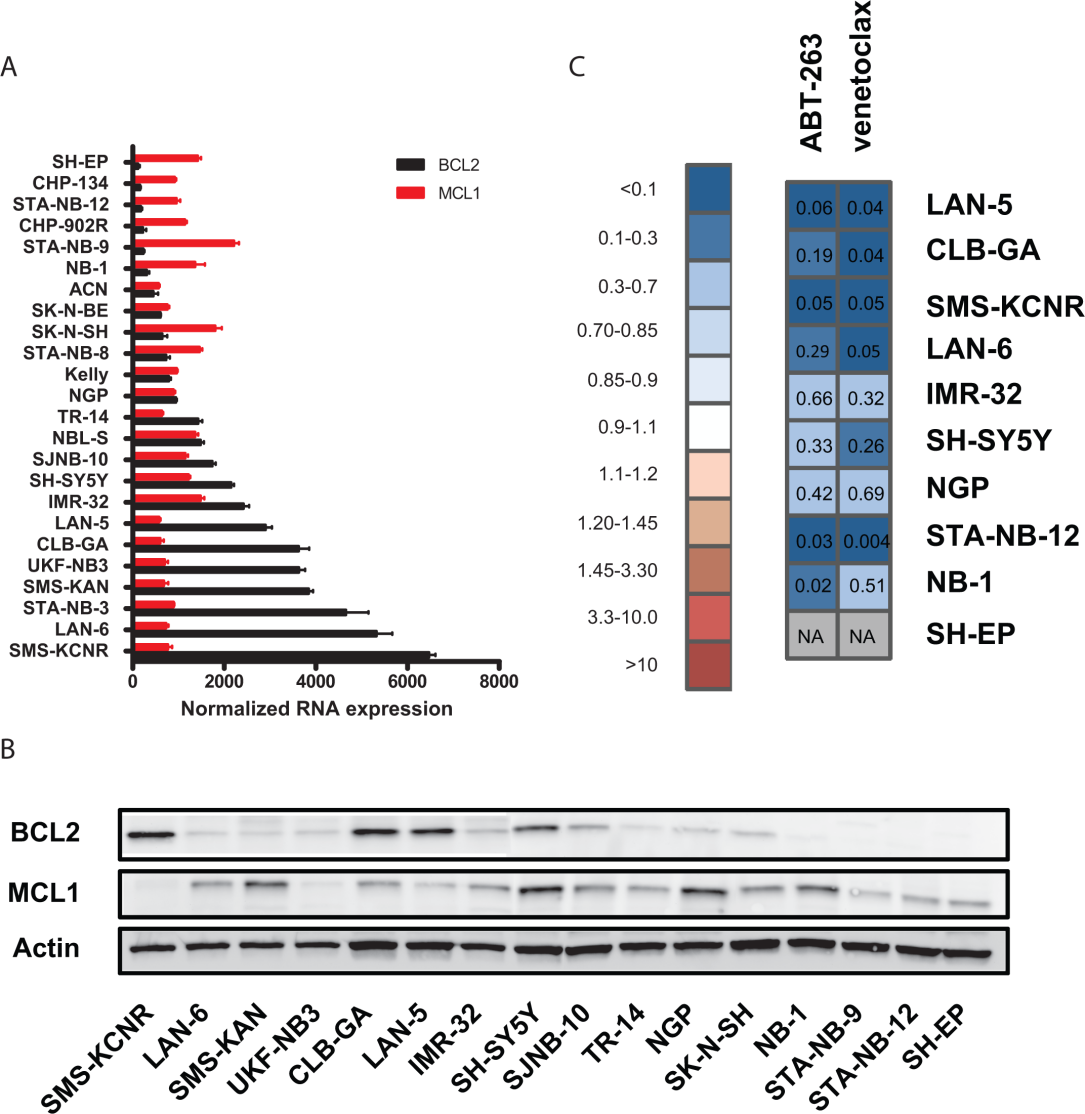
(**A**) BCL2 and MCL1 expression measured with RT-qPCR in a panel of neuroblastoma cell lines. Columns represent the normalized relative expression values and error bars represent the corresponding standard error. (**B**) Immunoblot analysis of BCL2 and MCL1 in neuroblastoma cell lines with β-actin as loading control. (**C**) Heatmap with CI values determined 24 hours after treatment with idasanutlin combined with venetoclax or ABT-263 in neuroblastoma cells. Depicted CI values represent the average of the CIs at ED50, ED75 and ED90 of at least two independent experiments. > 10: very strong antagonism; 3.30–10: strong antagonism; 1.45–3.30: antagonism; 1.20–1.45: moderate antagonism; 1.10–1.20: slight antagonism; 0.90–1.10: nearly additive; 0.85-0.90: slight synergism; 0.70–0.85: moderate synergism; 0.30-0.70: synergism; 0.10–0.30: strong synergism; < 0.10: very strong synergism.

BCL2 is an anti-apoptotic member of the BCL2 protein family and is often highly expressed in neuroblastoma tumors. BCL2 and MCL1 expression levels are correlated with the efficacy of BCL2 antagonists in neuroblastoma cells [22, 23]. Therefore, we determined BCL2 and MCL1 RNA and protein expression levels by RT-qPCR and Western Blotting, respectively, in a panel of neuroblastoma cell lines (Figure 2A, 2B). As described previously, we observed that neuroblastoma cells express either BCL2, MCL1 or both [22]. Moreover, we found discordance between BCL2/MCL1 protein and RNA expression levels, pointing to posttranscriptional regulation mechanisms. In order to assess whether combined treatment of ABT-263 and idasanutlin could have a broad therapeutic applicability in neuroblastoma, we evaluated both compounds in 10 human neuroblastoma cell lines with varying MCL1 and BCL2 expression levels. We treated cells for 24 hours with idasanutlin, ABT-263 or both and determined cell viability and CI values. With exception of the SH-EP cell line, that has a known inherent resistance to p53 activation, we found that the ABT-263/idasanutlin combination is highly synergistic in all evaluated neuroblastoma cell lines, with CI values ranging from 0.03 to 0.66 (Figure 2C). The occurrence of synergism was found independent of MCL1 and BCL2 expression levels, but higher drug concentrations had to be used in cells with low BCL2 expression levels to achieve therapeutic effect, which is in line with previous reports demonstrating that the efficacy of BCL2 antagonists is dependent on BCL2 expression levels [22, 23]. A mechanism-based thrombocytopenia due to BCL-XL binding has prevented the clinical development of ABT-263 and has stimulated the development of venetoclax, an analogue with negligible binding capacity for BCL-XL [24–26]. We therefore decided to evaluate the combinatorial treatment in our cell line panel with idasanutlin and venetoclax (Figure 2C). For all studied cell lines, we observed similar CI values when combining either venetoclax or ABT-263 with idasanutlin, with CI values between 0.004 and 0.69 indicative of strong synergism.

### Combined venetoclax/idasanutlin treatment enhances induction of apoptosis

**Figure 3:**
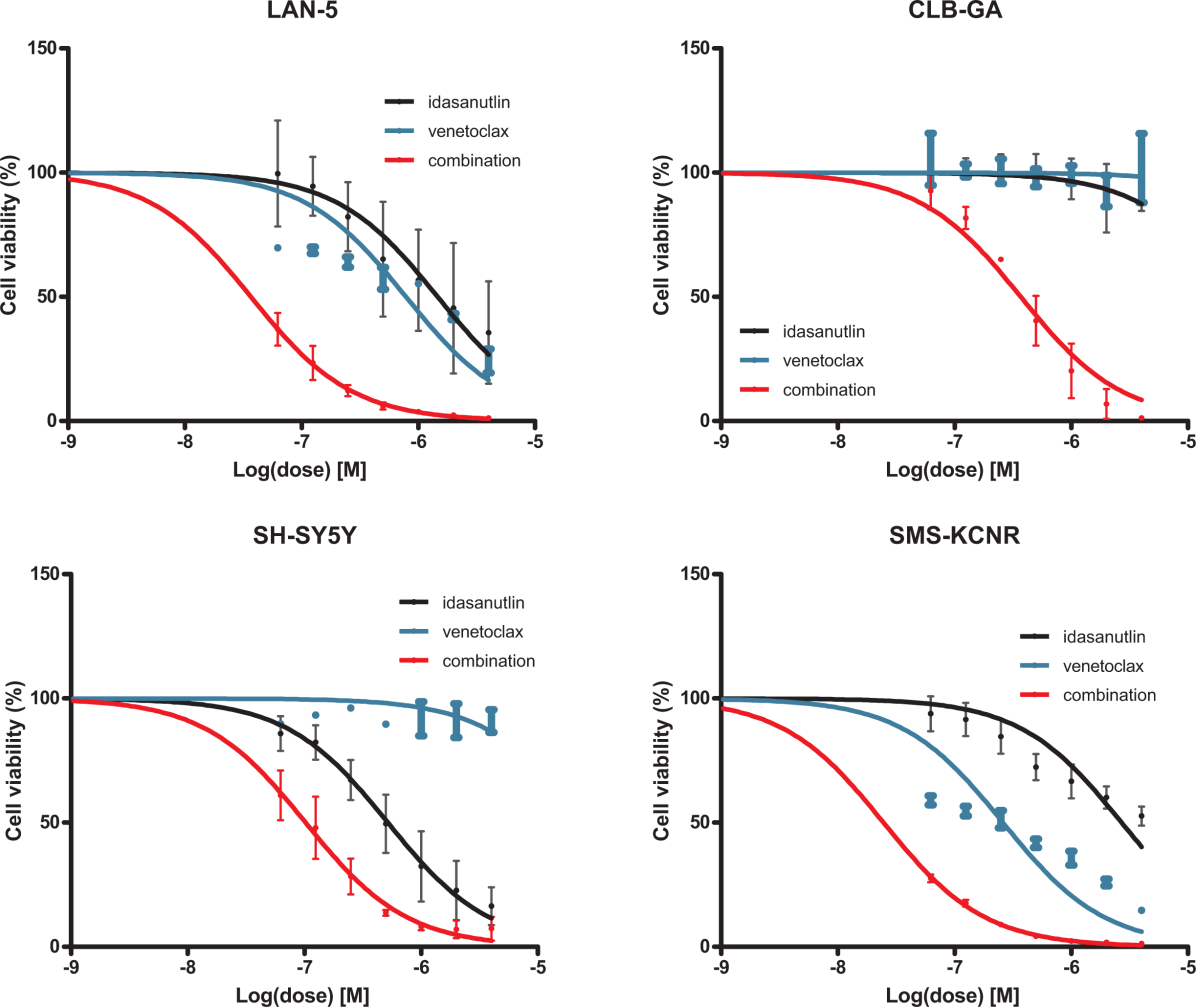
Effect of idasanutlin, venetoclax or both on neuroblastoma cell viability Exponentially growing neuroblastoma cells with high BCL2 expression (LAN-5, SMS-KCNR, CLB-GA and SH-SY5Y) were exposed to a range of idasanutlin and venetoclax concentrations for 24 hours and the percentage cell viability with respect to vehicle-treated controls was determined. Points represent the average of two independent experiments, each done in duplicate. Error bars represent the standard deviation.

**Figure 4:**
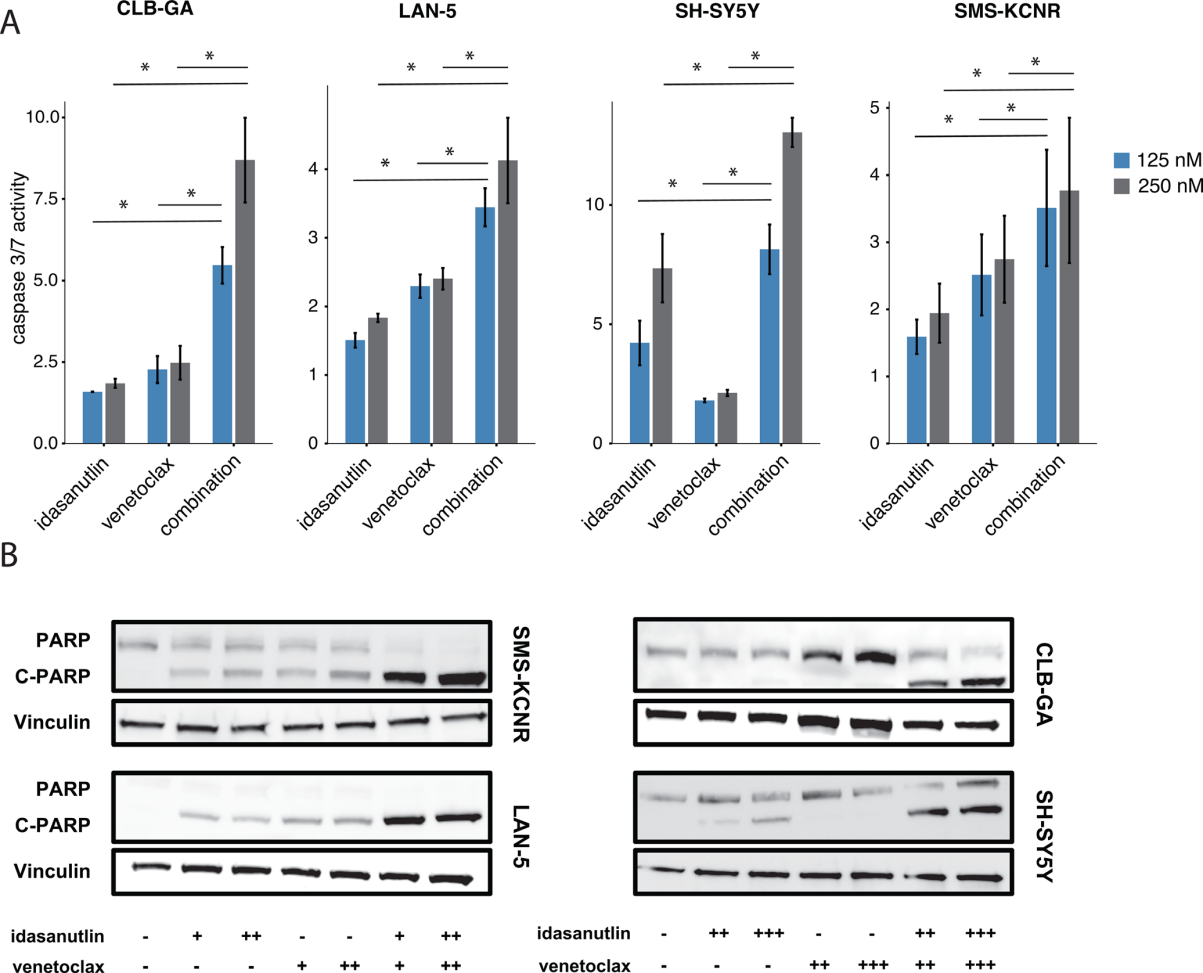
(**A**) Combined caspase-3 and caspase-7 activity in SH-SY5Y, SMS-KCNR, LAN-5 and CLB-GA cells after treatment with 125 nM or 250 nM of idasanutlin and/or venetoclax for 24 hours relative to vehicle-treated control cells. Caspase-3/7 activity was higher when cells were treated with venetoclax and idasanutlin as compared to each compound alone. Columns represent the average of two different experiments, each done in duplicate. Caspase-3/7 activity of the idasanutlin-venetoclax combination was compared to idasanutlin and venetoclax alone using the Mann–Whitney test. *p < 0.05. Error bars represent the standard deviation. (**B**) Immunoblot analysis of PARP cleavage in SH-SY5Y, SMS-KCNR, LAN-5 and CLB-GA cells after treatment with either 62.5 nM (+), 125 nM (++), or 250 nM (+++) of idasanutlin and/or venetoclax for 24 hours. Cleavage of PARP, a hallmark of apoptosis, was evident after combined idasanutlin / venetoclax treatment in all four cell lines. Vinculin was used as a loading control.

**Figure 5:**
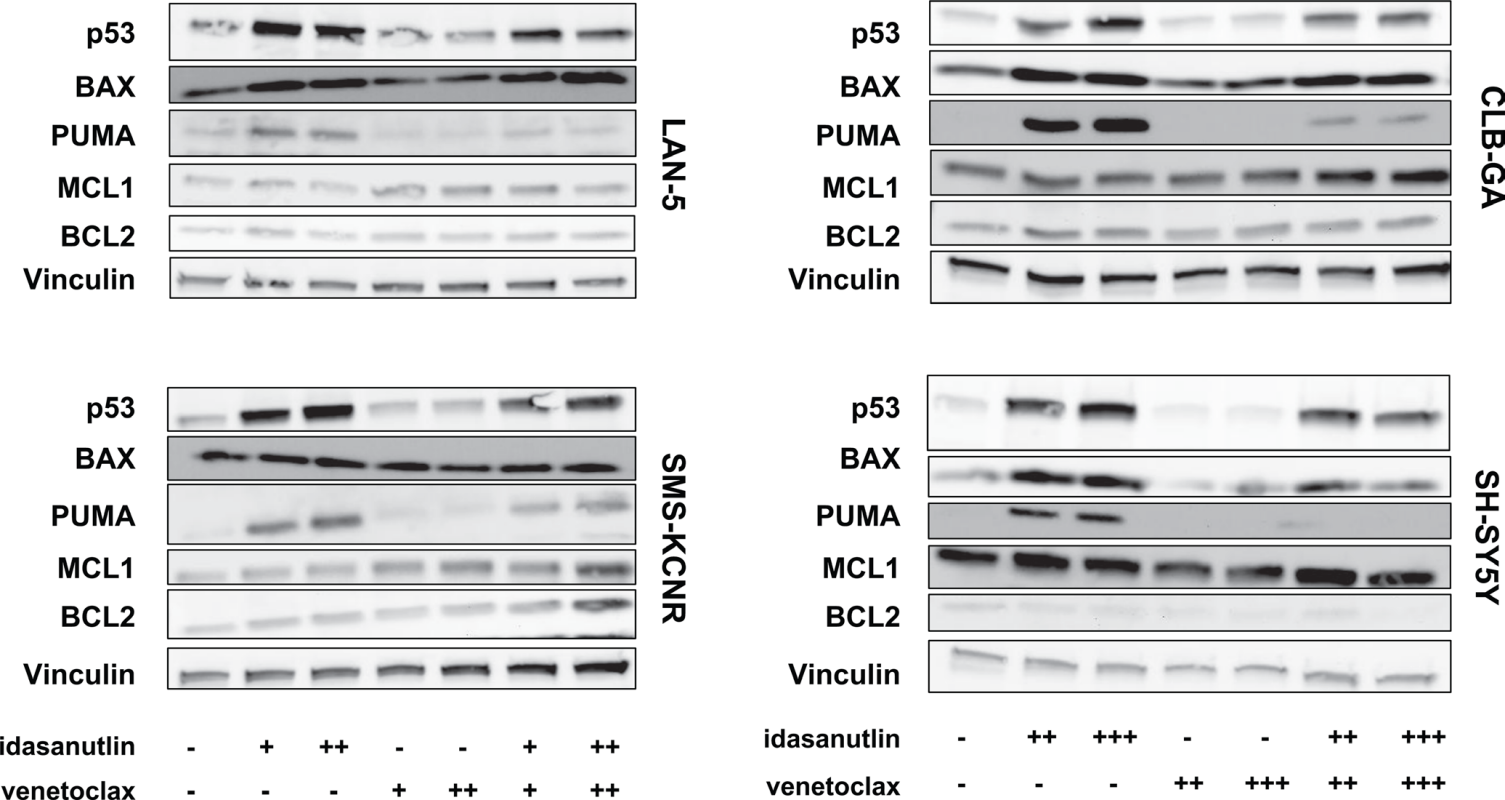
Immunoblot analysis of proteins involved in the p53 pathway and apoptosis SH-SY5Y, SMS-KCNR, LAN-5 and CLB-GA cells were treated with either 62.5 nM (+), 125 nM (++), or 250 nM (+++) of idasanutlin and/or venetoclax for 24 hours. After 24 hours of treatment, the protein abundance of p53, BAX, PUMA, MCL1 and BCL2 was determined by immunoblot analysis. Vinculin was used as loading control.

As the majority of neuroblastoma tumors is dependent on high BCL2 expression levels for survival, we evaluated the effects of combined venetoclax/idasanutlin treatment on cell viability and apoptosis in neuroblastoma cell lines with high BCL2 expression levels. Cell viability was determined 24 hours after SMS-KCNR, LAN-5, SH-SY5Y and CLB-GA cells were treated with idasanutlin, venetoclax or a combination of both (Figure 3). In all four cell lines the combination therapy dramatically reduced cell viability compared to each treatment alone. According to the proposed mechanism of action, combined treatment of idasanutlin and venetoclax should potently induce apoptosis [27]. In keeping with this hypothesis, we observed a significantly higher increase in caspase-3 and caspase-7 activity when combining both compounds as compared to single compound exposure in all cell lines (Figure 4A). Similar results were observed when PARP cleavage was assessed using Western blotting (Figure 4B). Next, we evaluated the protein levels of PUMA and BAX, two downstream mediators of p53-induced apoptosis and of the anti-apoptotic proteins MCL1 and BCL2, 24 hours after SMS-KCNR, LAN-5, SH-SY5Y and CLB-GA cells had been treated with idasanutlin, venetoclax or a combination of both (Figure 5). We observed increased protein abundance of pro-apoptotic PUMA and BAX upon idasanutlin treatment in all four neuroblastoma cell lines. BCL2 protein abundance was unaffected by treatment with either of the two drugs, with the exception of SMS-KCNR cells in which we observed higher BCL2 levels after treatment with the combination of both drugs. In addition, and in contrast to previous reports in acute myeloid leukemia cells [28], we did not observe a downregulation of MCL1 protein levels upon treatment with idasanutlin.

### Dual targeting of BCL2 and MDM2 inhibits neuroblastoma tumor growth *in vivo*

**Figure 6:**
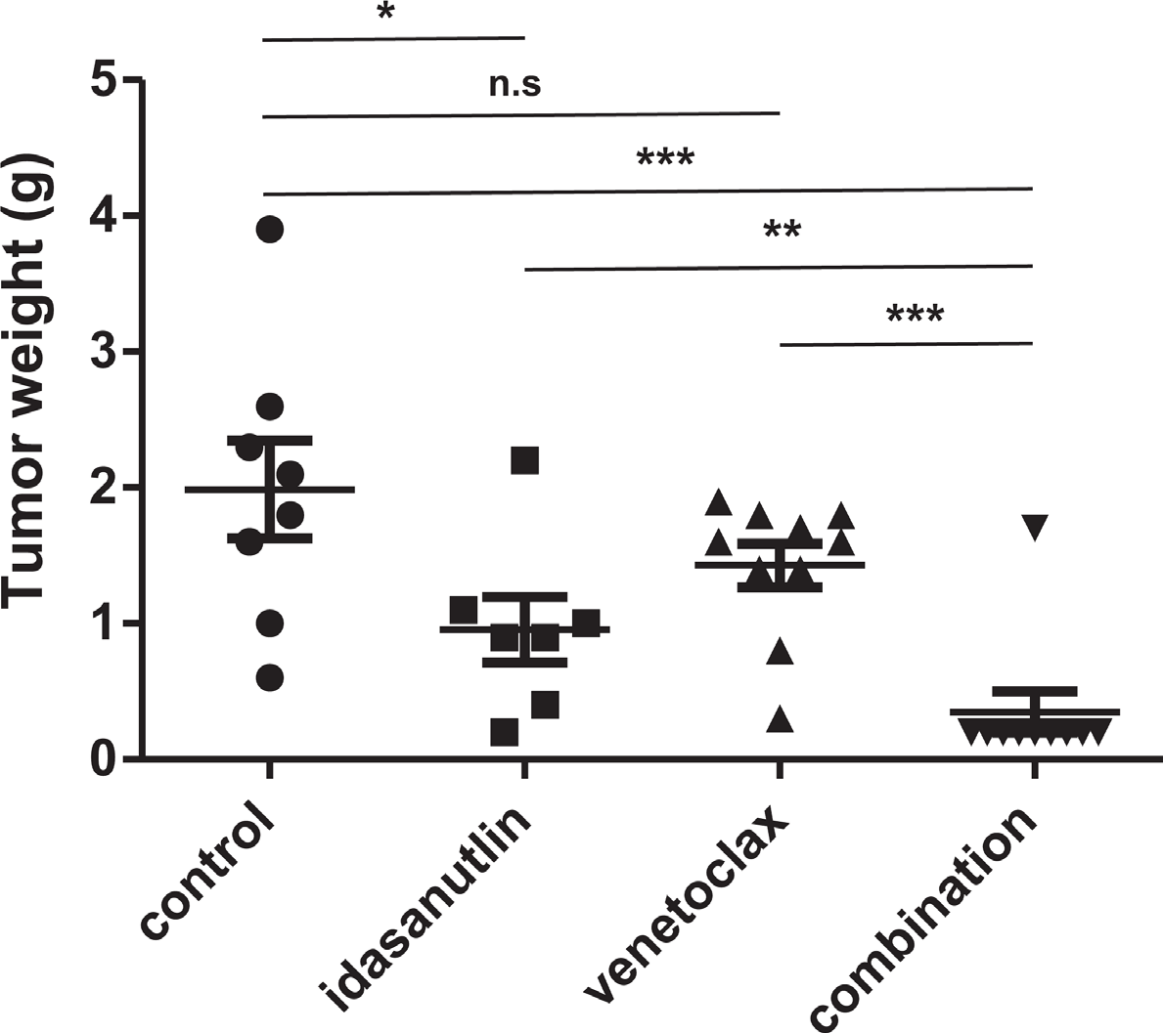
Tumor weights after 3 weeks of treatment with idasanutlin, venetoclax or both Nude mice carrying orthotopic xenografts of human SH-SY5Y/luc cells were treated by oral gavage with vehicle control, 75 mg/kg idasanutlin, 75 mg/kg venetoclax or both drugs, once daily, six times a week, for three weeks. Tumor weights were compared using the Mann–Whitney test. *p < 0.05, **p < 0.01, ***p < 0.001, n.s: not significant.

Despite a high degree of homology between murine and human MDM2, MDM2 antagonists have reduced binding affinity for mouse MDM2 in comparison to its human counterpart [15]. This interspecies selectivity hampers the use of murine transgenic models for the *in vivo* evaluation of MDM2 antagonists and prompted us to use a well-established model system utilizing orthotopic neuroblastoma xenografts. This model closely resembles the growth characteristics of primary neuroblastoma arising from the para-adrenal location in humans [29]. For the evaluation of the idasanutlin/venetoclax combination, nude mice carrying orthotopic xenografts of human SH-SY5Y/Luc cells were treated by oral gavage with vehicle control, 75 mg/kg idasanutlin, 75 mg/kg venetoclax or both drugs once daily, six times a week. After three weeks of treatment, mice were sacrificed and tumors were collected and weighed. When comparing with the control condition, we observed a significant reduction in tumor weight for mice treated with idasanutlin (*p* < 0.05, Mann–Whitney test) but not for mice treated with venetoclax (Figure 6). In mice treated with vehicle control, the average tumor weight was found to be 1.98 g (*n* = 8), compared to 0.95 g (*n* = 7) in mice treated with idasanutlin and 1.43 g (*n* = 10) in mice treated with venetoclax (Figure 5). This observation is in line with our *in vitro* findings (Figure 3). Most importantly, tumor weights of mice treated with both idasanutlin and venetoclax were found to be drastically lower than tumor weights of mice treated with either compound alone (*p* < 0.01 and *p* < 0.001 when comparing with idasanutlin and venetoclax respectively, Mann–Whitney test), with an average tumor weight of 0.35 g (*n* = 10). Mouse weights, possibly indicative of toxicity, were significantly lower in mice treated with idasanutlin (*p* < 0.05, Mann–Whitney test), but no significant difference was observed between the monotherapy and combination groups suggesting no additive adverse effects of the drug combination (Supplementary Figure 1).

To confirm these findings in an independent model system, we established orthotopic xenograft tumors of SMS-KCNR/luc human neuroblastoma cells in nude mice and administered 75 mg/kg idasanutlin or 50 mg/kg venetoclax or the combination of both treatments or vehicle control once daily by oral gavage for two weeks. Tumor weights at the end of the treatment period were on average 0.8 g (*n* = 9) in mice treated with the combination treatment regimen and 2.08 g (*n* = 6) in vehicle-treated mice, which reflected a significantly stronger tumor growth inhibition than after treatment with either drug alone (*p* < 0.05 and *p* < 0.01 when comparing with idasanutlin and venetoclax, respectively; Mann–Whitney test; Supplementary Figure 2). In conclusion, these *in vivo* data confirm our *in vitro* findings and strongly support further exploration of combined inhibition of MDM2 and BCL2 in the context of neuroblastoma.

## DISCUSSION

Small molecule MDM2 antagonists represent a potential novel therapeutic strategy for neuroblastoma treatment. Nutlin-3 is a cis-imidazoline analogue that specifically targets the hydrophobic p53-binding pocket of MDM2 and has shown prominent efficacy against p53^wt^ neuroblastoma cells both *in vitro* as *in vivo* [16]. RG7112, a nutlin drug with improved pharmacological properties provided biomarker-based evidence of clinical activity in a proof-of-mechanism trial in adult patients with well-differentiated or dedifferentiated liposarcoma [30]. Two recent preclinical studies have shown marked anti-neuroblastoma activity of idasanutlin, a second-generation MDM2 antagonist with superior potency and selectivity that is now prioritized for clinical development [19, 31]. Due to the development of resistance mechanisms, single-agent activity may however not prove sufficient to achieve sustained clinical efficacy of MDM2 antagonists [14]. Therefore, combination treatment of MDM2 antagonists with other drugs eliciting strong synergistic effects could result in more durable therapy responses and lower relapse rates while avoiding toxicity. Preclinical investigation of the combination of idasanutlin with chemotherapeutic agents in neuroblastoma has recently put forward temozolomide and busulfan as well-suited chemotherapeutic combination partners for idasanutlin [19]. In this study we aimed to provide a rational framework for combining idasanutlin with small molecule drugs.

We consistently found a high degree of synergism when combining idasanutlin with the BH3-mimetic ABT-263 in p53^wt^ cell lines and obtained similar results with venetoclax, a specific BCL2 inhibitor not targeting BCL-XL to safeguard against on-target thrombocytopenia. BCL2 is an anti-apoptotic member of the BH3 family and is highly expressed in the majority of neuroblastoma tumors. The efficacy of BCL2 inhibition has been extensively demonstrated using both *in vitro* and *in vivo* model systems of neuroblastoma [22, 23, 32–34]. In these studies, high endogenous levels of MCL1 have been reported to contribute to resistance to the cancer killing potential of BH3 mimetics. In contrast to the efficacy of monotherapy, we found that the venetoclax/idasanutlin combination is synergistic independent of MCL1 or BCL2 expression levels, suggesting a wide applicability in neuroblastoma. In neuroblastoma, nutlin-induced apoptosis is at least partially mediated by transcriptional activation of pro-apoptotic BCL2 family members like BAX and PUMA [7]. Apoptosis can be viewed as a process that is initiated when an apoptotic threshold is crossed as a result of BCL2 or MCL1 inhibition. BH3 mimetics lower the apoptotic threshold owing to their interaction with these anti-apoptotic BCL2 family members and could in this way potentiate p53-induced apoptosis [27]. Here, we observed elevated levels of apoptosis when combining venetoclax and idasanutlin compared to each treatment alone, as evidenced by increased PARP cleavage and increased caspase-3/7 activity, suggesting similar kinetics are present in neuroblastoma.

Interestingly, it has been reported that treatment of neuroblastoma cells with venetoclax can lead to increased levels of MCL1, resulting from a decreased expression of NOXA and increased stabilization by BIM [32]. Increased expression of MCL1 is a known resistance factor against treatment with BCL2 antagonists in various cancer types, including neuroblastoma [35]. Moreover, knockdown of MCL1 combined with venetoclax treatment results in synergistic growth inhibition of neuroblastoma cells [32, 36]. Since in acute myeloid leukemia the combination of idasanutlin with venetoclax results in an accelerated downregulation of MCL1 and subsequent induction of apoptosis [28], the same might hold true in neuroblastoma, providing an alternative explanation for the synergistic effects of this drug combination. We however observed increased protein levels of the apoptotic mediators PUMA and BAX upon idasanutlin treatment of neuroblastoma cells, but no downregulation of MCL1 protein or any effects on BCL2 protein abundance. Since we evaluated protein levels in neuroblastoma cell lines with high BCL2 expression levels and not in MCL1-dependent cell lines, it cannot be excluded that MCL1 is downregulated by idasanutlin treatment in the latter. Given these considerations, it might be interesting to determine the exact molecular mechanism in distinct types of neuroblastoma cell lines and to evaluate whether combined treatment with idasanutlin and venetoclax could overcome high MCL1 expression in an MCL1-inducible or venetoclax-resistant model system of neuroblastoma.

In conclusion, this study demonstrates both *in vitro* and *in vivo* that idasanutlin and venetoclax have highly synergistic anti-tumor effects in neuroblastoma. It is our hope that the results presented in this study may contribute to the development of clinically relevant targeted treatment schemes for this childhood tumor.

## MATERIALS AND METHODS

### Cell culture and compound treatment

Neuroblastoma cell lines used in this study (NGP, IMR-32, SH-SY5Y, SK-N-BE(2c), LAN-5, CLB-GA, SMS-KCNR, LAN-6, NB-1, STA-NB-12, SH-EP, STA-NB-3, SMS-KAN, UKF-NB3, SJNB-10, NBL-S, TR-14, Kelly, STA-NB-8, SK-N-SH, SK-N-BE, ACN, STA-NB-9, CHP-902R, CHP-134) were grown as monolayers in RPMI 1640 supplemented with 10% FCS, antibiotics and glutamine and maintained at 37°C and 5% CO_2_ in a humidified atmosphere. All cell lines used were genotyped to verify their identity. The following compounds were used: idasanutlin (kindly provided by Roche), venetoclax (Selleck Chemicals), ABT-263 (Selleck Chemicals), BI6727 (Selleck Chemicals), bortezomib (Selleck Chemicals), crizotinib (Sigma Aldrich), fenretinide (Tocris Bioscience), JQ1 (Gentaur), lestaurtinib (Sigma Aldrich), MLN8237 (Selleck Chemicals), PD332991 (Bio-Connect), rapamycin (Sigma-Aldrich), SJ172550 (Tocris Bioscience), tenovin-6 (Cayman Chemicals), U0126 (Sigma Aldrich), vorinostat (Sigma Aldrich), YM155 (Selleck Chemicals). A stock solution was made in either ethanol or DMSO and kept at −20°C. Cells were exposed to the indicated concentrations of compound for 24 hours with concentrations of DMSO and ethanol kept constant.

### Cell viability assays

Cells were plated at 80% confluency in duplicate wells in 96-well plates, incubated for at least 6 hours to permit adherence and treated with the indicated compounds for 24 hours. Cell viability was measured using the CELLtiter GLO Luminescent Cell Viability assay (Promega) according to the manufacturer’s instructions. At least two independent experiments were performed.

### Antibodies and western blotting

Cells were lysed using RIPA buffer with protease and phosphatase inhibitor cocktail (Roche). Protein concentration was quantified using the BCA method and immunoblotting was performed according to standard procedures. Proteins were fractionated using 10% SDS polyacrylamide gels (Bio-Rad) and transferred to a nitrocellulose membrane (Bio-Rad). The following primary antibodies were used: TP53 (#OP43, 1:1000) from Millipore, PUMA (#4976S, 1:1000), MCL1 (#5453S, 1:1000), cleaved PARP (#9546, 1:2000), BCL2 (#2872, 1:1000), vinculin (#4650P, 1:10000) from Cell Signaling, BAX (#2774P, 1:2000) from Merck Chemicals. The following secondary antibodies were used: anti-rabbit horseradish peroxidase (HRP, #7074S, 1:5000) and anti-mouse HRP (#7076S, 1:5000) from Cell Signaling. Proteins were visualized using Super Signal West Dura Extended Duration Substrate (Perbio Science).

### Apoptosis assays

For quantification of apoptosis, cells were plated in 96-well plates at 80% confluency, incubated for at least 6 h and exposed for 24 h to the indicated compound concentrations. Combined activity of caspase-3 and caspase-7 was determined using the Caspase-3/7 Glo assay (Promega) and measured with the Glomax multi detection system (Promega). At least two independent experiments were performed.

### Calculation of combination index (CI) values

Cell viability was determined as described above using a constant-ratio design. At least two independent experiments were performed. Cell viability values were converted to relative compound effect values and the CI values were calculated using Calcusyn software v.2 (Biosoft) according to Chou & Talalay (1984) [37]. Depicted CI values represent the average of the CI values at effective dose 50, 75 and 90 (ED50, ED75 and ED90).

### Orthotopic xenograft model

Orthotopic neuroblastoma xenografts were generated in four to six week old female athymic immunodeficient (Nu/Nu) mice as previously described [29]. Briefly, 1 × 10^6^ human SH-SY5Y/luc or SMS-KCNR/luc neuroblastoma cells were surgically implanted beneath the renal capsule encompassing the renal gland and kidney. Approximately two weeks after implantation, tumor establishment was confirmed by bioluminescent luciferase imaging and treatment was started. Mice carrying tumors of SH-SY5Y/luc cells were treated with 75 mg/kg idasanutlin (dissolved in hydroxypropylcellulose/Tween-80), 75 mg/kg venetoclax (dissolved in 60% Phosal, 30 % PEG and 10 % ethanol) or both by oral gavage on a daily basis, six times a week, for a total of three weeks. Mice carrying tumors of SMS-KCNR/luc cells were treated with 75 mg/kg idasanutlin (dissolved in hydroxypropylcellulose/Tween-80), 50 mg/kg venetoclax (dissolved in 60% Phosal, 30% PEG and 10% ethanol) or both by oral gavage on a daily basis, five times a week, for a total of two weeks. Idasanutlin and venetoclax were kindly provided by Roche and AbbVie, respectively. After treatment, mice were sacrificed and tumors were collected and weighed. All experimental protocols were approved by the Ethical Committee of Baylor College of Medicine and carried out in accordance with the relevant guidelines and regulations.

## SUPPLEMENTARY MATERIALS FIGURES AND TABLES


