# Supplementary material for: Dual targeting of MDM2 and BCL2 as a therapeutic strategy in neuroblastoma
Source: Oncotarget. 2017 Jul 4;8(34):57047–57. doi: 10.18632/oncotarget.18982 (PMC5593624; doi:10.18632/oncotarget.18982)
Supplement: Supplementary file 1 [file oncotarget-08-57047-s001.pdf]

Dual targeting of MDM2 and BCL2 as a therapeutic strategy in neuroblastoma

SUPPLEMENTARY MATERIALS

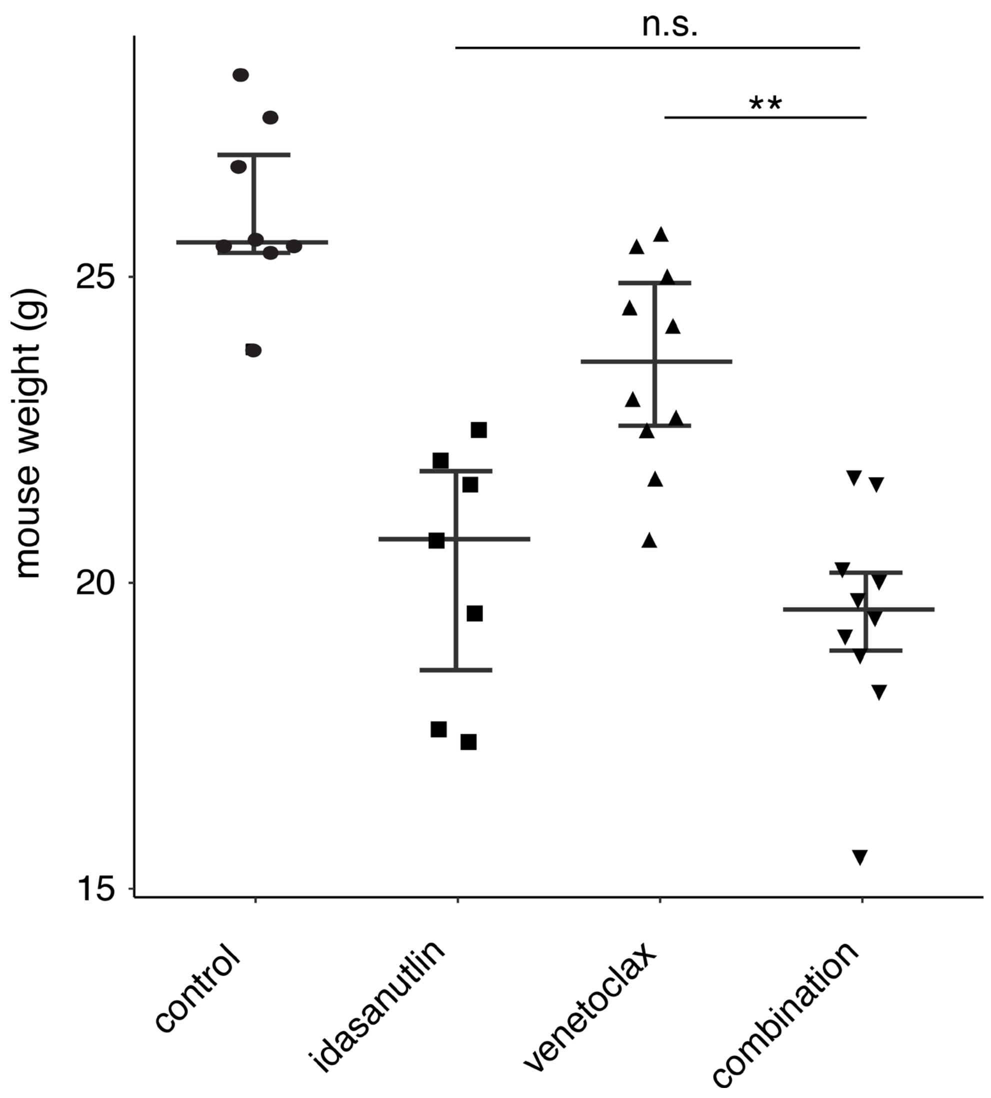

Supplementary Figure 1: Mouse weights in grams after 3 weeks of treatment with idasanutlin, venetoclax or both. Nude mice carrying orthotopic xenografts of human SH-SY5Y cells were treated by oral gavage with vehicle control, 75 mg/kg idasanutlin, 75 mg/kg venetoclax or both drugs, once daily, six times a week, for three weeks. Mouse weights were compared using the Mann-Whitney test. **:$p < 0.01$, n.s.: not significant.

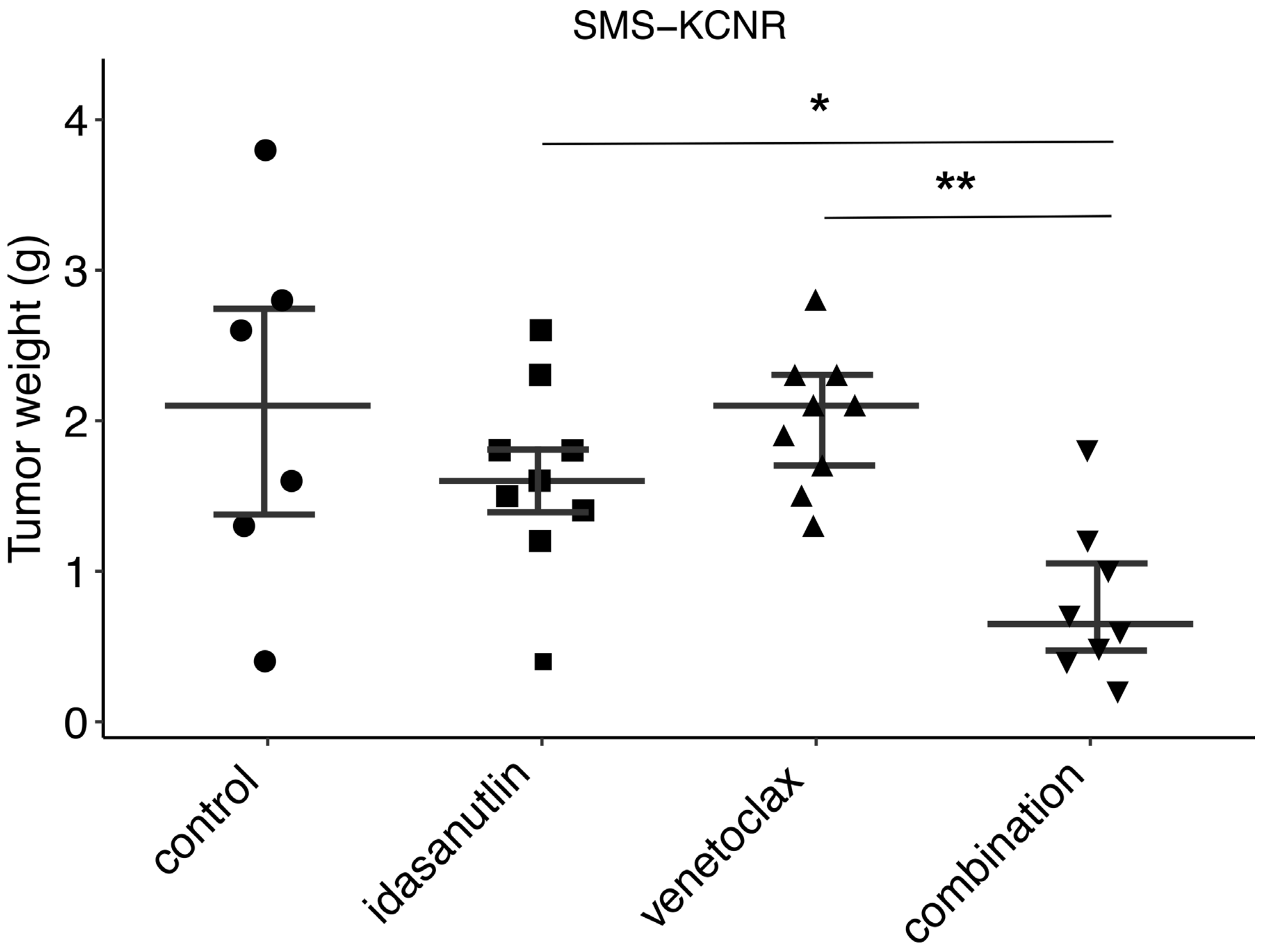

Supplementary Figure 2: Tumor weights in grams after 2 weeks of treatment with idasanutlin, venetoclax or both. Nude mice carrying orthotopic xenografts of human SMS-KCNR/luc cells were treated by oral gavage with vehicle control, 75 mg/kg idasanutlin, 50 mg/kg venetoclax or both drugs, once daily, five times a week, for two weeks. Tumor weights were compared using the Mann-Whitney test. *$p < 0.05$, **$p < 0.01$.

Supplementary Table 1: Schematic overview of the different drugs used in combination with idasanutlin and their respective targets and dose ranges (nM)

Drug	Target	Dose range (nM)
RG7388	MDM2	0–4800
ABT-263	BCL2	0–1600
YM155	survivin	0–1600
JQ1	c-MYC/MYCN through BRD4	0–32000
Crizotinib	c-Met/ALK	0–3200
MLN8237	AURKA	0–16000
Fenretinide	synthetic retinoid	0–8000
Lestaurtinib	multi-kinase inhibitor with potent anti-Trk activity	0–3200
U0126	MEK1/2	0–16000
Rapamycin	mTOR	0–40000
PD-33291	CDK4/6	0–1200
Tenovin-6	SIRT1/2	0–16000
Vorinostat	HDAC	0–5600
BI6727	PLK1	0–1600
Bortezomib	proteasome	0–1600
SJ172550	MDM4	0–32000

Supplementary Table 2: Cell viability data of SK-N-BE(2c) measured 24h after treatment with idasanutlin, ABT-263 or a combination of both. Values represent the mean of at least two biological replicates +/− the standard deviation

Concentration (nM)		Cell viability (%)	Cell viability (%)	Cell viability (%)
idasanutlin	ABT-263	idasanutlin	ABT-263	combination
0	0	100 +/− 4	100 +/− 9	100 +/− 6
250	100	102 +/− 4	91 +/− 6	97 +/− 6
500	200	99 +/− 3	92+/− 7	96 +/− 4
1000	400	100 +/− 3	96 +/−6	90 +/− 10
2000	800	92 +/− 4	94 +/− 9	86 +/− 10
4000	1600	96 +/− 8	94 +/− 7	90 +/− 8
